# Case Report: Congenital tuberculosis in a premature infant requiring extracorporeal membrane oxygenation

**DOI:** 10.1051/ject/2023007

**Published:** 2023-06-28

**Authors:** Hugh K. Quach, Briana L. Scott, Denise A. Lopez-Domowicz, Rachel M. Gambino, Amy E. Evans, Caroline P. Ozment

**Affiliations:** 1 Department of Pediatrics, Division of Critical Care Medicine, University of Alabama at Birmingham, Children’s Hospital of Alabama Birmingham AL 35233 USA; 2 Department of Pediatrics, Division of Critical Care Medicine, Duke University Medical Center Durham NC 27710 USA; 3 Department of Pediatrics, Division of Critical Care Medicine, St. Vincent Mercy Medical Center Toledo OH 43608 USA; 4 Perfusion Services, Duke University Medical Center Durham NC 27710 USA

**Keywords:** Congenital tuberculosis, Extracorporeal membrane oxygenation, *Mycobacterium bovis*

## Abstract

Congenital tuberculosis is a rare infectious disease with less than 500 cases documented worldwide. Mortality is significant, ranging from 34 to 53%, and death without treatment is inevitable. Patients exhibit nonspecific symptoms such as fever, cough, respiratory distress, feeding intolerance, and irritability which can make appropriate diagnosis challenging in Peng et al. (2011) Pediatr Pulmonol 46(12), 1215–1224. Tuberculosis prevalence is particularly high in developing countries where access to resources can be limited in World Health Organization (2019) Global tuberculosis report 2019, Geneva. We present a 2.4-kg premature male infant with acute respiratory distress syndrome secondary to congenital tuberculosis caused by *Mycobacterium bovis* and tuberculosis-immune reconstitution inflammatory syndrome who was successfully supported with veno-arterial extracorporeal membrane oxygenation.

## Overview

Globally, tuberculosis (TB) is a top ten cause of death – killing approximately 1 million people annually. Ten million new TB cases occur worldwide with 66% in developing countries. In the United States, 8900 cases were reported in 2019. TB infection includes pathogens that cause mycobacterium tuberculosis complex, including *Mycobacterium tuberculosis, Mycobacterium bovis,* and *Mycobacterium africanus*. Though *Mycobacterium tuberculosis* is the most frequently identified, the other two species can also cause systemic disease and present similarly. The World Health Organization estimated 140,000 cases of TB caused by *M. bovis* occurred in 2019 [1].

TB may have various presentations and can result in respiratory failure – with isolated pulmonary disease or systemic disease. In severe disease, patients may require extracorporeal membrane oxygenation (ECMO) support. These cases have been reported in both adults and children. Monier et al. present a rare case of miliary TB with histiocytic hemophagocytosis in a 14-year-old female requiring ECMO support for 144 h. She made a full recovery after decannulation and appropriate anti-TB therapy [2]. Petrillo et al. presented a 15-year-old female with isolated respiratory failure and delayed diagnosis of TB who also survived following 152 h of ECMO [3]. Complicating presentations further, with appropriate treatment, patients may exhibit worsening symptoms due to immune reconstitution inflammatory syndrome (IRIS) which is a paradoxical excessive immune response to TB complexes [4].

The aforementioned cases represent acquired TB as adolescents. Another presentation entirely is congenital TB. Congenital TB is an extremely rare disease, which is challenging to identify and difficult to treat. Disease transmission occurs vertically despite the placenta acting as a natural barrier. Hematogenous spread from the placenta to the fetal liver [5] and/or by fetal swallowing of infected amniotic fluid into the lungs contributes to bacterial dissemination [6]. After a congenital infection, prior studies suggest typical disease presentation in the first 3 weeks of life, though in some cases it may be months prior to diagnosis. This may be because congenital TB presents with non-specific symptoms, and is often asymptomatic in mothers and fetuses in the perinatal period [5]. It has been reported that these patients may later develop significant respiratory failure, shock, and multiorgan dysfunction/failure and carries a mortality rate of 34–53% [7]. As such, these patients may require ECMO support to survive.

In this case report, we present the second reported case of congenital TB requiring ECMO [8] and the first reported case of congenital tuberculosis caused by *M. bovis*.

## Description

A 27-year-old G1P0 immigrant Guatemalan woman with a history of infertility requiring *in vitro* fertilization presented in preterm labor after a primarily uneventful prenatal course. She gave birth to a 1.465 kg male infant at 29 weeks and 6 days gestation who was admitted to the neonatal intensive care unit (NICU), placed on continuous positive airway pressure (CPAP) for respiratory distress, and started on caffeine for presumed apnea of prematurity. The patient received routine care in the NICU for the first three weeks of life but was unable to wean off CPAP. During the fourth week of life, the patient had increasing apnea/bradycardia/desaturation (ABD) events and required increasing respiratory support. Chest X-ray demonstrated bilateral alveolar and interstitial opacities suggestive of developing pneumonia or acute respiratory distress syndrome (ARDS). The patient was started on empiric antibiotics with ampicillin, gentamicin, and azithromycin to include coverage for atypical pneumonia. An infectious work-up, including blood and urine cultures, respiratory viral panel, herpes simplex virus panel, and gonorrhea/chlamydia probe was unremarkable. However, the patient did have a mildly elevated CRP. Antibiotics were subsequently discontinued, and the clinical picture was attributed to possible aspiration given the concomitant escalation of enteral nutrition at the time of decompensation.

**Figure 1:**
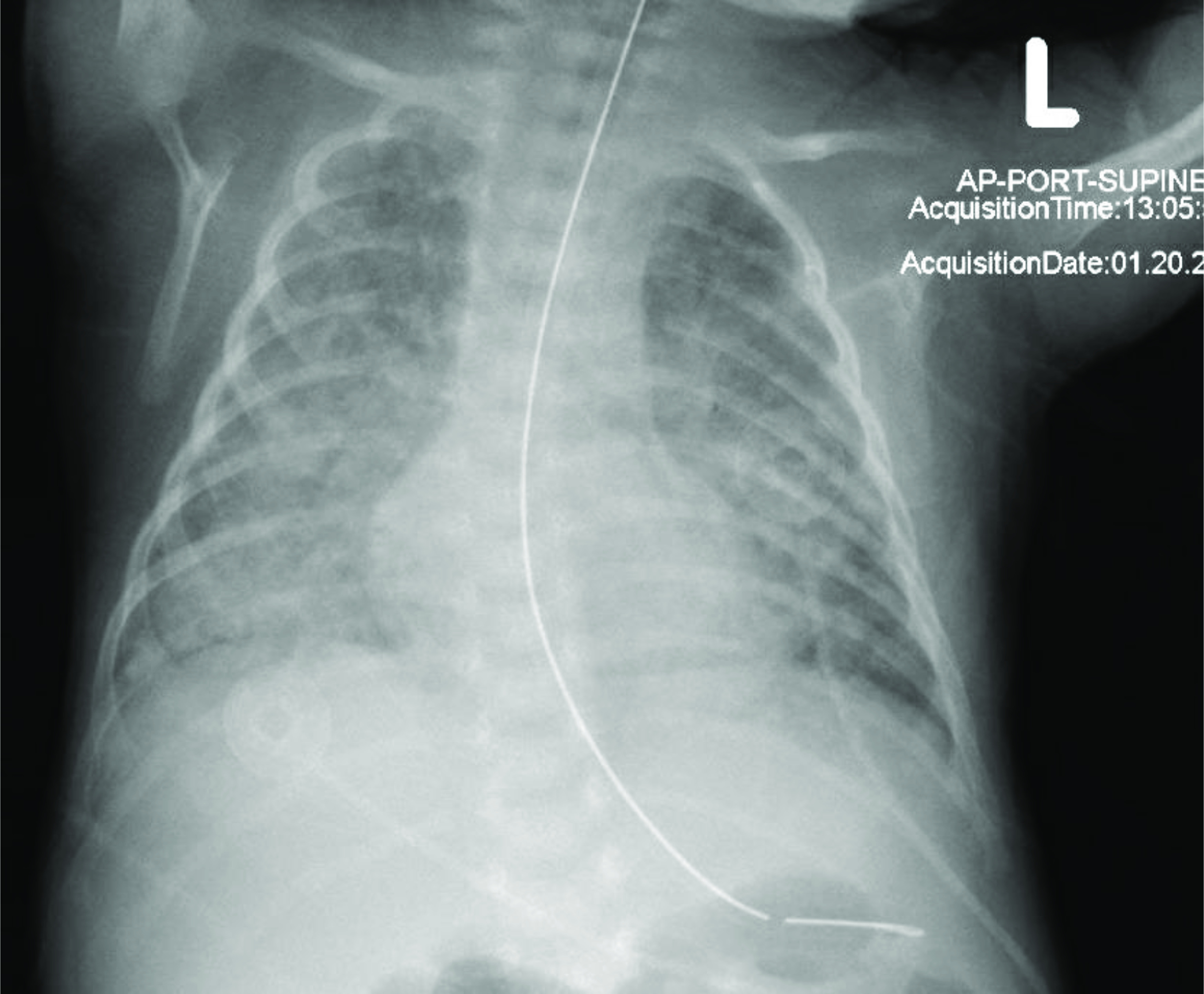
Chest X-ray with diffuse alveolar and interstitial opacities, *R* > *L*, possibly representative of developing pneumonia/ARDS.

The patient continued to require increasing respiratory support and was started on dexamethasone to prevent bronchopulmonary dysplasia. Chest radiographs showed persistent, severe interstitial disease and the CRP continued to rise (Figure 1). At 5 weeks of age, the patient became more lethargic, hypoxic, and hypercarbic and was ultimately intubated. At that time, the patient received surfactant and was restarted on empiric antibiotics. One week later, prompted by an infectious disease consultation, a further infectious work-up was initiated with the addition of both potassium hydroxide staining and acid-fast bacilli (AFB) staining from endotracheal sputum culture.

**Figure 2:**
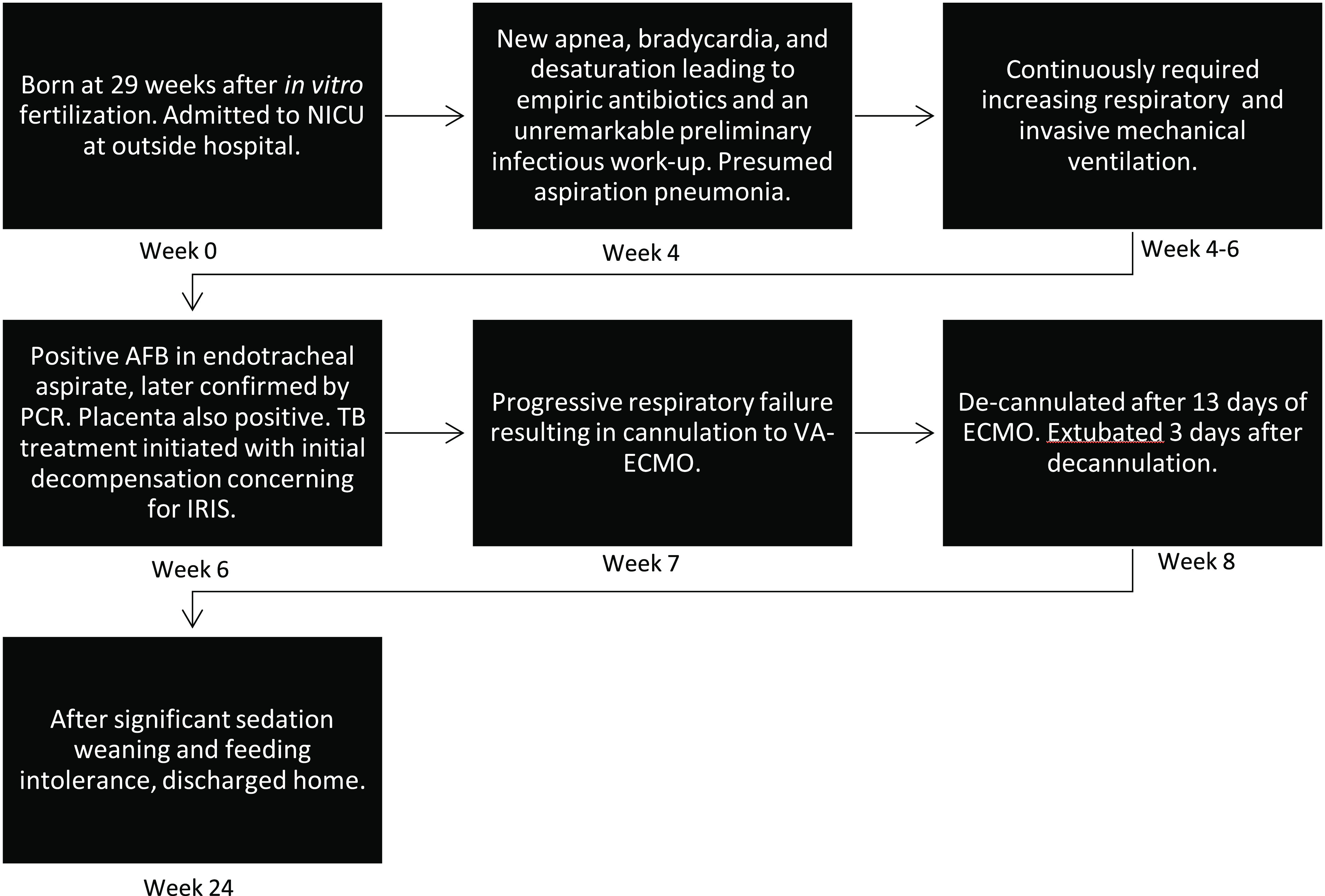
Timeline depicting clinical course of patient case.

Two days later, at 40 days of life, AFB staining was positive, and subsequent *Mycobacterium tuberculosis* complex was detected by polymerase chain reaction (PCR), confirming tuberculosis infection. Treatment for TB was initiated with pyrazinamide, isoniazid, rifampin, levofloxacin, and vitamin B6. The mother remained asymptomatic, although further investigation showed that placental microscopic staining was positive for AFB and she was diagnosed with urogenital TB – confirming the patient’s diagnosis of congenital TB (Figure 2).

With the initiation of antibiotics, the patient began experiencing hypotension and required increasing respiratory support leading to continuous infusions of epinephrine and dopamine at 6 weeks of age and weighing 2.4 kg, the patient was transferred to this institution for ECMO cannulation and support. Physical exam at that time was unremarkable except for crackles and rhonchi on auscultation. Within hours of arrival, the patient acutely decompensated despite high ventilator settings and was subsequently cannulated onto ECMO after discussion with and consent from the parents. Given the acute decompensation after starting anti-TB therapy, dexamethasone was continued for possible IRIS [4].

Given the patient’s vessel size, veno-arterial (VA) ECMO was pursued. The patient was placed under standard airborne precautions in the operating room with negative pressure conditions and the use of N95 masks and given systemic heparinization. A pediatric cardiothoracic surgeon performed a cutdown and an 8 French Biomedicus arterial cannula was placed in the right common carotid artery (Medtronic, Minneapolis, MN), and a 10 French Biomedicus venous cannula was placed in the right internal jugular vein (Medtronic, Minneapolis, MN). There were no mechanical or bleeding complications during cannulation. ECMO support was provided with a Maquet CardioHelp console utilizing a CardioHelp 5.0 Bioline pump and oxygenator (Getinge, Gothenburg, Sweden), custom ¼″ arteriovenous (AV) loop with a continuous manifold shunt (LivaNova, London, United Kingdom), air/oxygen blender (Sechrist, Anaheim, CA), and a Micro-Temp heater (Cincinnati Sub-Zero, OH, USA). An ultrasonic flow probe verified patent ECMO flow distal to all shunts.

ECMO flow was initiated at 100 mL/kg/min and ventilator settings were weaned to standard institutional ECMO rest settings. Vasopressors were discontinued and aggressive diuresis was initiated with furosemide and chlorothiazide. The patient was anticoagulated with a continuous heparin infusion per institutional protocol to an activated partial thromboplastin time (aPTT) of 80–100 s. A robust native cardiac ejection in the setting of poor lung function resulted in relatively deoxygenated blood being supplied to the coronary arteries. Thus, the patient required relatively high ECMO flows, up to 140 mL/kg/min, to shunt more blood away from the heart and into the ECMO circuit to better ensure oxygenated perfusion to the myocardium. Following this, the patient had a relatively uncomplicated ECMO course with no issues with forward cardiac flow, significant hemolysis, or bleeding. Over the course of 13 days, the patient was weaned to a sweep of 0.5 LPM, FiO_2_ of 70%, and 25 mL/min of CO_2_ was bled in with ventilator settings of PIP 19, PEEP 6, and FiO_2_ of 30%. At this time, the ECMO circuit was cycled down sequentially to 100 mL/kg/min, then 75 mL/kg/min, and ultimately 35 mL/kg/min until a clamp trial was initiated. With each of these changes, patient hemodynamics were continuously observed, and arterial blood gases were obtained every 3–5 min. Due to the stability of these metrics, the patient was decannulated and three days later the patient was successfully extubated.

Four days following extubation, the patient had a respiratory arrest secondary to aspiration and required re-intubation for 3 weeks. During this time, endotracheal tube samples underwent genotyping and were positive for *Mycobacterium bovis*.

After a prolonged hospital course complicated by sedation weaning and feeding intolerance, the patient was discharged at six months of life. The patient, fortunately, did not develop other complications of congenital TB such as bone marrow suppression or altered liver function. On follow-up at seven months of life, the patient continued to have feeding difficulties requiring a gastrostomy tube but was no longer requiring oxygen support and was meeting 4-month developmental milestones.

## Discussion

Congenital TB is rare, and while likely under-recognized and under-reported there are less than 500 documented cases. It is endemic to countries with limited resources, which contributes to its significant mortality rate of 34–53% [7]. We discuss the second reported case of congenital TB requiring ECMO [1] and the first reported case of congenital TB secondary to *M. bovis*.

This patient presented with non-specific symptoms that can mimic other disease processes common in premature neonates – making early diagnosis difficult. Pneumonia and aspiration were both suspected causes of the non-specific apnea/bradycardia/desaturation events before ultimately arriving at the diagnosis of congenital TB at 6 weeks of life. Further, the mother was asymptomatic, and her diagnosis of urogenital TB was made retrospectively after the placental AFB staining and PCR confirmed the presence of the mycobacterium tuberculosis complex. Interestingly, after anti-TB treatment was initiated the patient experienced hypotension and clinical compromise. This seems to indicate that the patient might have been experiencing TB-IRIS.

Outside of this difficulty in diagnosis, another challenge was navigating through the patient’s medical history. The patient’s father spoke traditional Spanish but the mother, the primary bedside guardian, spoke a Guatemalan dialect which was a barrier to communication. As such, it was difficult to ascertain the mother’s personal medical history and it was through some later interviews that it was determined the likely source of *Mycobacterium bovis* infection was from consuming unpasteurized dairy products. Given this and the nonspecific symptoms of congenital TB, clinicians may want to consider the diagnosis of congenital TB in children presenting similarly if the patient or parents are from an endemic area.

While congenital TB is largely a respiratory disease process, this patient was cannulated to VA-ECMO due to vessel size limitations. In a retrospective study of 39 neonates placed on VA-ECMO rather than VV-ECMO for respiratory failure, the indication for VA-ECMO was patient size limitation in 10% of patients [9]. Despite the known advantages of VV-ECMO, which include decreased risk of cerebral events, reduced hypoxic pulmonary vasoconstriction, and preservation of the carotid artery, recent literature reports approximately 75% of neonates underwent VA-ECMO cannulation for respiratory failure [10, 11]. Furthermore, when peripheral VA-ECMO is utilized in the setting of persistent respiratory failure, differential hypoxia may occur, which is well-described in the context of femoro-femoral VA-ECMO for adults. Similarly, in infants, when VA-ECMO via the carotid artery is used for respiratory failure, a type of V/Q mismatch may occur if the patient has a significant native cardiac ejection relative to the ECMO flows. Compromised pulmonary function results in deoxygenated blood returning to the left atrium and subsequently being ejected from the left ventricle [12]. Despite 125 mL/kg of VA-ECMO flow for the patient, the significant native cardiac output resulted in a systemic PaO_2_ of 47 mmHg, after which point the ECMO flow was increased to 140 mL/kg/min. In doing so, the ratio of hypoxic native ejection to the oxygenated ECMO flow was reduced, resulting in improved PaO_2_.

Additionally, for the ECMO circuit of this patient, an adult CardioHelp oxygenator was utilized. The use of the CardioHelp in pediatrics is rarely discussed in the literature [13] but has been the standard practice of this institution for pediatric/neonatal patients for nearly 10 years. Prior to using the Cardiohelp, circuits at this institution included centrifugal pumps like the Sorin Revolution and the Quadrox D adult oxygenator. The practice was then transitioned to Cardiohelp due to ease of priming, safety profile, smaller footprint, and decreased battery consumption. An adult-sized oxygenator is utilized since it is the only type that has a polymethyl pentene membrane on the market. The neonatal/pediatric Quadrox was previously trialed, but this oxygenator anecdotally encountered issues with obstruction, rapid rises in transmembrane pressure, and early circuit failure.

This patient was decannulated after ECMO and was successfully extubated and discharged. Though the patient had a long hospitalization, the patient represents the second successful case of ECMO utilization in congenital TB and the first report of ECMO rescue for *M. bovis-*associated ARDS. This may lend to the future use of ECMO in patients with congenital TB as migrant populations grow in non-endemic areas and as ECMO becomes more available in endemic areas.

## Data Availability

No new data were created or analyzed in this study.
